# Performance Feedback Processing Is Positively Biased As Predicted by Attribution Theory

**DOI:** 10.1371/journal.pone.0148581

**Published:** 2016-02-05

**Authors:** Christoph W. Korn, Gabriela Rosenblau, Julia M. Rodriguez Buritica, Hauke R. Heekeren

**Affiliations:** 1 Department of Education and Psychology, Freie Universität Berlin, Berlin, Germany; 2 Department of Psychiatry, Psychotherapy, and Psychosomatics, University of Zurich, Zurich, Switzerland; 3 Neuroscience Center Zurich, University of Zurich, Zurich, Switzerland; 4 Center for Translational Developmental Neuroscience, Child Study Center, Yale University, New Haven, CT, United States of America; 5 Berlin School of Mind and Brain, Humboldt-Universitaet zu Berlin, Berlin, Germany; Centre national de la recherche scientifique, FRANCE

## Abstract

A considerable literature on attribution theory has shown that healthy individuals exhibit a positivity bias when inferring the causes of evaluative feedback on their performance. They tend to attribute positive feedback internally (e.g., to their own abilities) but negative feedback externally (e.g., to environmental factors). However, all empirical demonstrations of this bias suffer from at least one of the three following drawbacks: First, participants directly judge explicit causes for their performance. Second, participants have to imagine events instead of experiencing them. Third, participants assess their performance only after receiving feedback and thus differences in baseline assessments cannot be excluded. It is therefore unclear whether the classically reported positivity bias generalizes to setups without these drawbacks. Here, we aimed at establishing the relevance of attributions for decision-making by showing an attribution-related positivity bias in a decision-making task. We developed a novel task, which allowed us to test how participants changed their evaluations in response to positive and negative feedback about performance. Specifically, we used videos of actors expressing different facial emotional expressions. Participants were first asked to evaluate the actors’ credibility in expressing a particular emotion. After this initial rating, participants performed an emotion recognition task and did—or did not—receive feedback on their veridical performance. Finally, participants re-rated the actors’ credibility, which provided a measure of how they changed their evaluations after feedback. Attribution theory predicts that participants change their evaluations of the actors’ credibility toward the positive after receiving positive performance feedback and toward the negative after negative performance feedback. Our results were in line with this prediction. A control condition without feedback showed that correct or incorrect performance alone could not explain the observed positivity bias. Furthermore, participants’ behavior in our task was linked to the most widely used measure of attribution style. In sum, our findings suggest that positive and negative performance feedback influences the evaluation of task-related stimuli, as predicted by attribution theory. Therefore, our study points to the relevance of attribution theory for feedback processing in decision-making and provides a novel outlook for decision-making biases.

## Introduction

The ambiguous nature of causality assessments and their relation to self-related positivity biases have long been acknowledged within the context of attribution theory, which ranks as one of the central psychological theories to describe motivated behavior in humans ([1,2]; see [3,4] for an overview of its historical development). Its main tenet states that future behavior depends on the evaluation of the causes for performance feedback (e.g., results of a mathematics exam), which is often dichotomized into conveying correct performance (or success) and incorrect performance (or failure) [2]. Potential causes are classified along three dimensions that span a matrix of possible causal explanations: i) locus: internal versus external, ii) stability: stable versus unstable, and iii) globality: global versus specific causes [5]. Some formulations of attribution theory focus on controllability as the third dimension [2]. Thus, negative feedback conveying incorrect task performance can be ascribed to multiple causes (e.g., lack of ability, task difficulty, or bad luck) and individuals may infer the most likely cause for negative feedback in a self-serving way [1,2].

### Causality attributions and motivational biases

Considerable research has shown that meta-cognitive assessments of causality create some leeway for motivational biases such that correct performance is ascribed to internal factors (e.g., one’s own ability) and incorrect performance is more likely attributed externally (e.g., task difficulty or bad luck) [6–8]. For example, students tend to ascribe their good performance in a mathematics exam to a high level of ability but their poor performance to an unjustifiably difficult test [9]. This classic self-related positivity bias, often referred to as the “self-serving bias”, is supposed to play an important, adaptive role for maintaining psychological well-being and mental health [6]. The opposite tendency, ascribing negative events to internal, stable, or global factors, is associated with a higher number of depressive symptoms [7,10].

### Established measures of causality attributions

The empirical assessments of attributional styles in healthy and clinical populations have vastly relied on explicit, verbal self-reports of inferred causality. Typically, participants are asked to write down and/or rate the most likely causes for hypothetical events, real life events, or performance feedback on an experimental task. The Attributional Style Questionnaire (ASQ) [5] and its variants constitute the most widely used self-report measure and assess the attribution of hypothetical events on the dimensions locus, stability, and globality. In a meta-analysis of the self-serving bias in attribution [7], 54% of the included effect sizes were based on variants of the ASQ and an additional 10% on similar measures (see Table 7 in [7]). The remaining 36% of the effect sizes were derived from studies that used some kind of experimental manipulation but all of them prompted participants to provide or rate explicit causes. Similarly, most neuroscientific studies on attribution used stimuli adapted from the ASQ [11–14], while some of them employed feedback on cognitive [15] or emotional [16] tasks but still asked participants to explicitly choose between different causal explanations. Undoubtedly, these explicit measures of attributional styles have the advantage of precisely translating attribution theory into empirical investigation. However, previous demonstrations of an attribution-related positivity bias suffer from at least one of three drawbacks.

### Drawbacks of established measures

The first and most important drawback is that explicit assessments of causality lack generalizability to real life situations. In most real life situations (for example the school setting), humans process feedback implicitly (i.e., spontaneously and without conscious effort or control) [17]. Implicit processes mediate different behavioral aspects than explicit processes [18] and can only be measured indirectly. To provide a striking example from another domain, a person with implicit racial prejudices might display a particular bias on an implicit task, without reporting prejudices when asked explicitly [19,20]. Therefore, implicit tasks have become more and more common especially in the domain of social decision-making (see [18,21] for reviews and [22] for a recent example).

A second drawback is that most assessments of causality involve hypothetical and/or loosely defined events, which the participants have to imagine (e.g., in the ASQ). Due to the ambiguous nature of hypothetical events (the underlying action-feedback contingencies are undefined), the exact triggers for a person’s attribution processes remain elusive.

A third drawback is that in most tasks participants are asked to provide a single rating for the relevant stimuli. Therefore, baseline differences that are independent of performance feedback cannot be excluded.

### Advantages of decision-making tasks

In contrast, studies in decision (neuro)science have used tasks that do not suffer from the drawbacks mentioned above to measure implicit attitudes, affective states, or information processing biases [23–26]. First, participants’ beliefs are mostly assessed in the form of implicit ratings or choices and not explicit verbal reports. Second, decision-making tasks use narrowly defined real life events and/or clear-cut feedback during the task itself. Third, decision-making tasks often repeatedly assess participants’ beliefs to investigate how they dynamically change due to obtained feedback. Negative feedback due to incorrect task performance, for example, should lead to greater efforts to improve performance in the future. Indeed, research in decision (neuro)science has made great strides in describing feedback processing, often with the help of computational models (e.g., reinforcement learning or drift-diffusion models) [27–32].

### Decision-making tasks and motivational biases

Crucially, dynamically assessing participants’ beliefs before and after self-relevant feedback has revealed self-related positivity biases in feedback processing. Healthy humans update their beliefs more after receiving desirable than after receiving undesirable information: When participants are provided with the true base rates of clearly defined adverse life events they adjust their beliefs more for desirable information (i.e., after initial overestimations) than for undesirable information (i.e., after initial underestimations) [33,34]. Relatedly, social feedback from peers on character traits leads to a greater change of participants’ self-evaluations when the feedback is desirable than when it is undesirable [35,36].

### Open questions

It is an open question whether the self-serving bias reported in the attribution literature (see [7] for a meta-analysis) emerges in a well-controlled setup akin to those commonly used in the decision-making literature (see [27–32] for reviews). More generally, it is unclear whether attributions are relevant for decisions since theories on decision-making typically neglect that performance feedback can have multiple causes and that the ascribed causes may taint future beliefs and decisions.

### Aims and operationalization

Based on attribution theory, we predicted a positivity bias in the processing of performance feedback. The aim of the current study was thus to establish the relevance of attribution theory for decision-making by testing whether humans show positively biased processing of performance feedback in a setup that controls for three drawbacks commonly present in investigations of attribution theory. Crucially, participants were not prompted to provide explicit statements of causality. Rather they were asked to state their beliefs about the employed stimuli before and after receiving performance feedback. We chose to operationalize performance feedback in an emotion recognition task that comprised dynamic facial emotional expressions. As dependent variables, we measured participants’ beliefs about the credibility of the depicted emotion before and after the emotion recognition task. Positively biased attributions were assessed as a “self-serving” change in belief: Incorrect performance should be attributed externally and thus result in a downgrading of the actor (“I performed incorrectly because the actor’s depiction of the emotion was not credible—not because of a lack of emotion recognition skills.”). In contrast, correct performance may lead to a more positive assessment of the actor.

### Implementation with emotional facial expressions

We used emotional facial expressions because of their high social saliency [37]. Emotional visual stimuli are detected faster [38–40] and recognized more accurately [41,42] than non-emotional visual stimuli, in particular when the faces have dynamic properties [43,44].

### Task overview and hypotheses

We used a within-subjects design with participants’ ratings as main dependent measure. Specifically, participants performed an emotion recognition task that included 96 video clips of various actors depicting different facial emotions. In half of the trials, participants received the veridical feedback on whether they correctly labeled the depicted facial emotion or not. In the other half of the trials, no feedback was provided. Crucially, participants rated the actors’ credibility in performing the facial emotions before and after the emotion recognition test. Thereby, we could measure how participants updated their assessment of credibility depending on task performance and performance feedback. We expected that participants would make updates toward the positive (i.e., higher second credibility rating) after feedback on correct task performance. After feedback on incorrect task performance, participants were expected to make updates toward the negative (i.e., lower second credibility rating). The condition, in which participants received no feedback, served as a control condition: We expected this differential updating to be absent or reduced when no performance feedback was provided. To explore how the behavioral pattern in our task may relate to classic measures of attribution, we correlated update scores with scores on the Attributional Style Questionnaire (ASQ).

## Methods

### Participants

**Fig 1:**
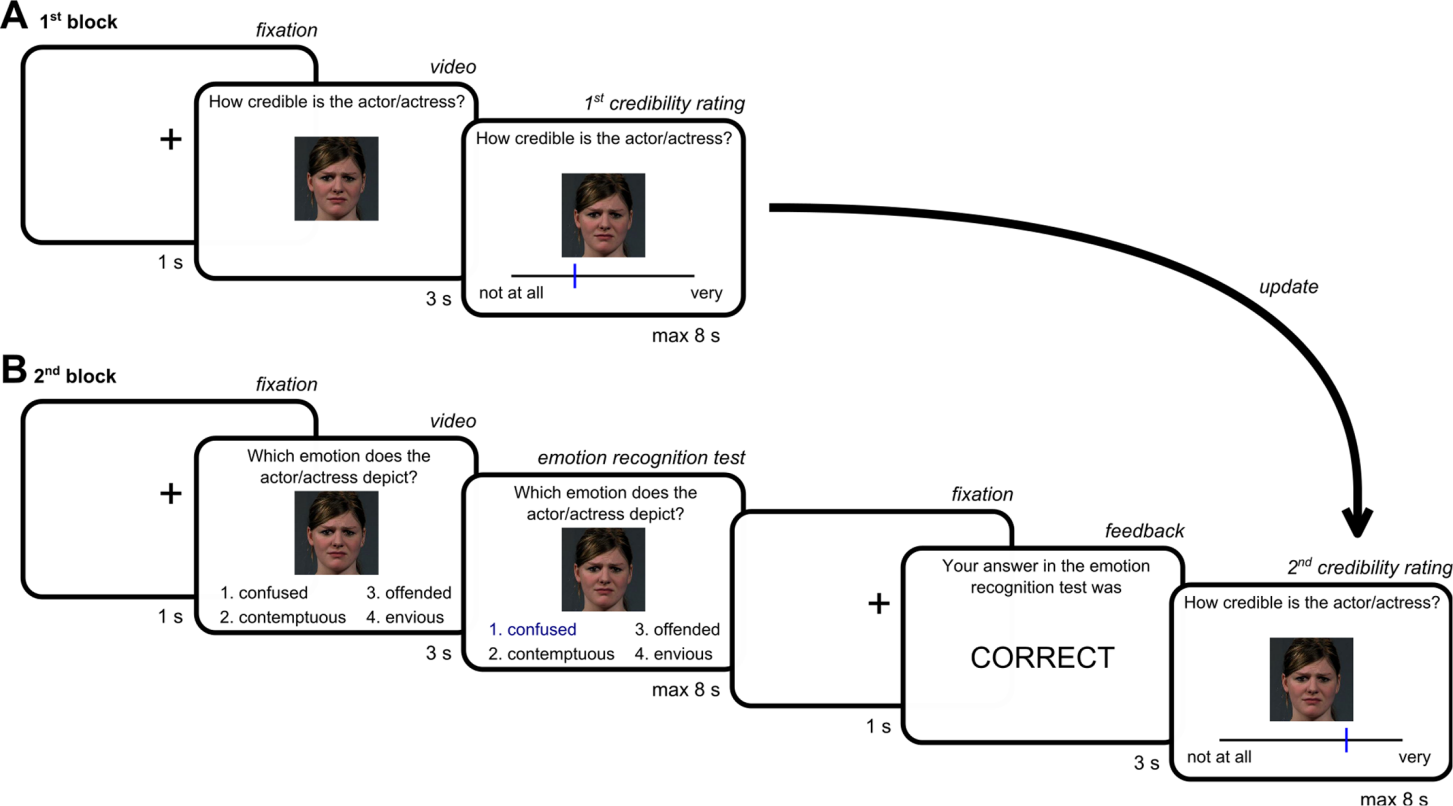
Task design. (A) In the first block, participants used a visual analogue scale to rate the credibility of different actors portraying different facial emotions. In each of the 96 trials, participants first saw a dynamic video and then were asked to provide their rating while the last frame of the video remained on screen as a still image. Each video item showed one of 26 actors depicting one of 23 different emotions. (B) In the second block, participants performed an emotion recognition task followed by a second credibility rating. The correct emotion label was presented along with three distractors. We selected videos and distractors so that overall task performance was around 50%. Immediately after the emotion recognition test, participants either received veridical performance feedback or no feedback (“XXXXXX”). Update scores were calculated as the difference between second and first credibility ratings.

Participants (n = 25, 20 female, mean age = 26, *SD* = 3.8) were recruited via flyers and mailing lists at the Freie Universität Berlin. They were paid 10 € for participating in the experiment, which took approximately one hour to complete. The study was approved by the ethics committee of the German Society for Psychology (DGPs). The individual depicted in Fig 1 in this manuscript has given written informed consent (as outlined in PLOS consent form) to publish these details.

### Experimental set-up

#### Task instructions

The experiment took place in the testing rooms of the Freie Universität Berlin under the supervision of a trained experimenter. Participants gave written informed consent to participate in the study and filled out a demographic questionnaire. Subsequently, they received a detailed written task instruction. To avoid potential biases, participants were not supposed to know the real objective of this study. The introduction provided an incentive for participants to perform well on the task. Participants were told that they would be presented with a standardized emotion recognition test, which had been previously normed in a population-based study. They were further told that, after the test, they would receive a report relating their performance to the average performance of their age-and gender-based comparison group. Prior to the test, participants were asked to rate the credibility of the actors portraying the emotions displayed in the test items.

#### First credibility rating

In the first block of the experiment, participants rated the credibility of actors portraying different facial emotions (see Fig 1A). We explained to participants that credibility ratings should capture how closely an actor’s portrayal resembled natural emotional expressions that participants encounter in everyday life. This, we told them, would provide us with an additional validity assessment of the emotion recognition test.

Participants were presented with 96 video items of 26 actors (13 female) depicting one of 23 different types of facial emotions (see Stimuli for details). For each video item, participants rated how credible the actors’ performance was on a visual analogue scale ranging from low to high credibility. Specifically, in each trial a fixation cross was presented (1 s) before the video clip (3 s). The last frame of the video remained on the screen as a still images when participants were prompted to rate the actors’ credibility using a slider, which had a resolution of 270 positions. The slider was adapted from one of our previous studies [45]. Participants moved the slider leftwards or rightwards using the corresponding arrow keys and confirmed their choices by pressing the “down” arrow key. If participants responded within 8 s, they saw a confirmation of their choice for 0.5 s; otherwise they saw the prompt “too slow” on the screen. Trial order was pseudo-randomized for each participant so that no emotion type and no actor occurred two times in a row.

#### Emotion recognition test and second credibility rating

In the second block, participants performed an emotion recognition test including all previously rated video items (see Fig 1B). We informed participants that during the emotion recognition test, they would sometimes receive feedback on whether or not they solved the items correctly, while other times they would not get feedback. Participants were asked to solve the emotion recognition task as quickly and accurately as possible. After solving each item, they would have to rate the credibility of the actor’s depiction of the facial emotion in the same fashion as they had done in the first block. Specifically, in each trial a fixation cross (1 s) was presented before the video clip (3 s) with four emotion adjectives (e.g., confused, contemptuous, offended, envious). The last frame of the video remained on the screen and participants had 8 s to indicate the correct adjective for the depicted emotion using the keys 1, 2, 3, or 4. Again participants saw the prompt “too slow” if they did not respond within the time frame of 8 s. After another fixation cross (1 s), participants received feedback on their task performance in half of the trials (3 s; “Your answer in the emotion recognition test was correct/incorrect”). In the other half of the trials, participants received no feedback (“XXXXXX”). Then they were asked to rate the actors’ credibility a second time using the slider as in the first block of the experiment. Again trial order was pseudo-randomized so that no emotion type and no actor occurred two or three times in a row. Within each trial the order of the emotion adjectives was randomized. For the assignment of the condition (feedback versus no feedback), the 96 video items were split into two lists (see Stimuli). The assignment of the two lists to the feedback versus no feedback condition was counterbalanced across participants. The experiment was presented using the software Presentation (Version 14.1, Neurobehavioral Systems Inc., Albany, CA).

### Attributional Style Questionnaire

After the experiment participants completed the Attributional Style Questionnaire (ASQ) [5,46]. The ASQ is a self-report questionnaire that classifies a person’s explanatory style for 12 hypothetical events (half bad and half good) using three causal dimensions: internal versus external, stable versus unstable, and global versus specific causes. Participants were asked to write down the one major cause of each event and then rate the cause along a 7-point continuum for each of the three causal dimensions (internality, stability, and globality). Completion of the questionnaire was self-paced. Two participants were not assessed on the ASQ. Two participants did not complete the ASQ.

Participants were debriefed after the experiment. They were informed about the real objective of the experiment and told that they would not receive a performance report, as the test was not standardized.

### Stimuli

To increase the ecological validity of the experiment, the 96 stimuli used in the emotion recognition test were video sequences (video duration: 3s) depicting 23 emotional facial expressions portrayed by 26 professional actors (13 female). We included 2 to 6 emotional expressions from each actor (see S1 Table). The included emotions were selected on the basis of a previous study that classified emotional words according to their valence and arousal as well as their communicative frequency in everyday life [47]. Compared to most established emotion recognition tests, our test comprised a larger number of different emotions, which also included basic emotions (e.g., angry, sad) that have been shown to involve universal, highly stereotypical physiological reactions [48,49]. For more information on stimulus production and stimulus validation procedure please refer to [50].

Prior to the study, we validated the task with 144 video items, portrayed by 28 actors (15 female), in a separate sample of 14 pilot participants (11 female, mean age = 24.6, *SD* = 2.74). Since our task design requires a splitting into correctly and incorrectly answered items, the pilot study served the purpose of excluding items with a floor or ceiling effect on correctness in the emotion recognition task. We excluded items, which were answered correctly by less than 14% or more than 93% participants in the pilot study. Additionally, we selected items so that the mean item correctness of the selected items was approximately 50%. A mean item correctness of around 50% ensures a balanced task design (50% of the times participants would get the feedback that they solved the item correctly or incorrectly).

Based on this criterion, we included 96 items in our final stimulus set. Participants in the main study solved on average correctly 49% (*SD* = 8) of the final items. The average credibility rating of the emotional expressions before the emotion recognition test was 49 (*SD* = 11) on a scale ranging from 1 to 100 (see below). The 96 items were divided into two item lists (corresponding to the two conditions of the experiment: feedback versus no feedback), each comprising 48 items. The lists were closely matched with respect to the actors’ age group, gender, valence, arousal and frequency of emotional expressions (see S1 Table) and were counterbalanced between conditions.

### Data analysis

Trials for which participants failed to respond in the allotted time of maximally 8s for the first or second ratings or the emotion recognition task were excluded from all analyses. Overall, participants missed on average 1.24 trials (*SD* = 1.88). Mean reaction times (RTs) for correct and incorrect performance in the emotion recognition task were compared using a paired *t*-test. For the ratings, RTs could not be analyzed because participants answered via a visual analogue scale with a slider that always started at the midpoint of the scale.

#### Credibility ratings and updates

All ratings were transformed from their original values on the visual analogue scale to values on a scale ranging from 1 to 100. Update scores were calculated as the difference between first and second ratings.

update=secondrating−firstrating

Thus, positive updates indicate an increase in credibility ratings (a change towards the positive) while negative updates indicate a decrease in credibility ratings (a change towards the negative). We compared mean update scores as well as mean first and second ratings per participant and condition using repeated measures ANOVAs with two within-subjects factors and follow-up *t*-tests.

#### Effects of the video items

To additionally account for the possible effects of the video items, linear mixed effects models were set up using the function lmer implemented in the lme4 package of the software R (www.r-project.org). Independent variables were the updating scores, first or second ratings of all answered trials for all individual participants. Fixed effects comprised predictors for Condition and Correctness as well as their interaction. We included random effects for participants and for video items, which included random intercepts and random slopes for the highest interaction (i.e., the Condition X Correctness interaction) [51]. This is equivalent to the following R model formula.

Y∼condition*correctness+(1+condition:correctness|participants)+(1+condition:correctness|items)

Significance levels of the fixed effects interactions were determined by performing log-likelihood tests, which compared the full model to a reduced model without the interaction factor.

## Results

### Mean update scores indicated a positivity bias following performance feedback

**Fig 2:**
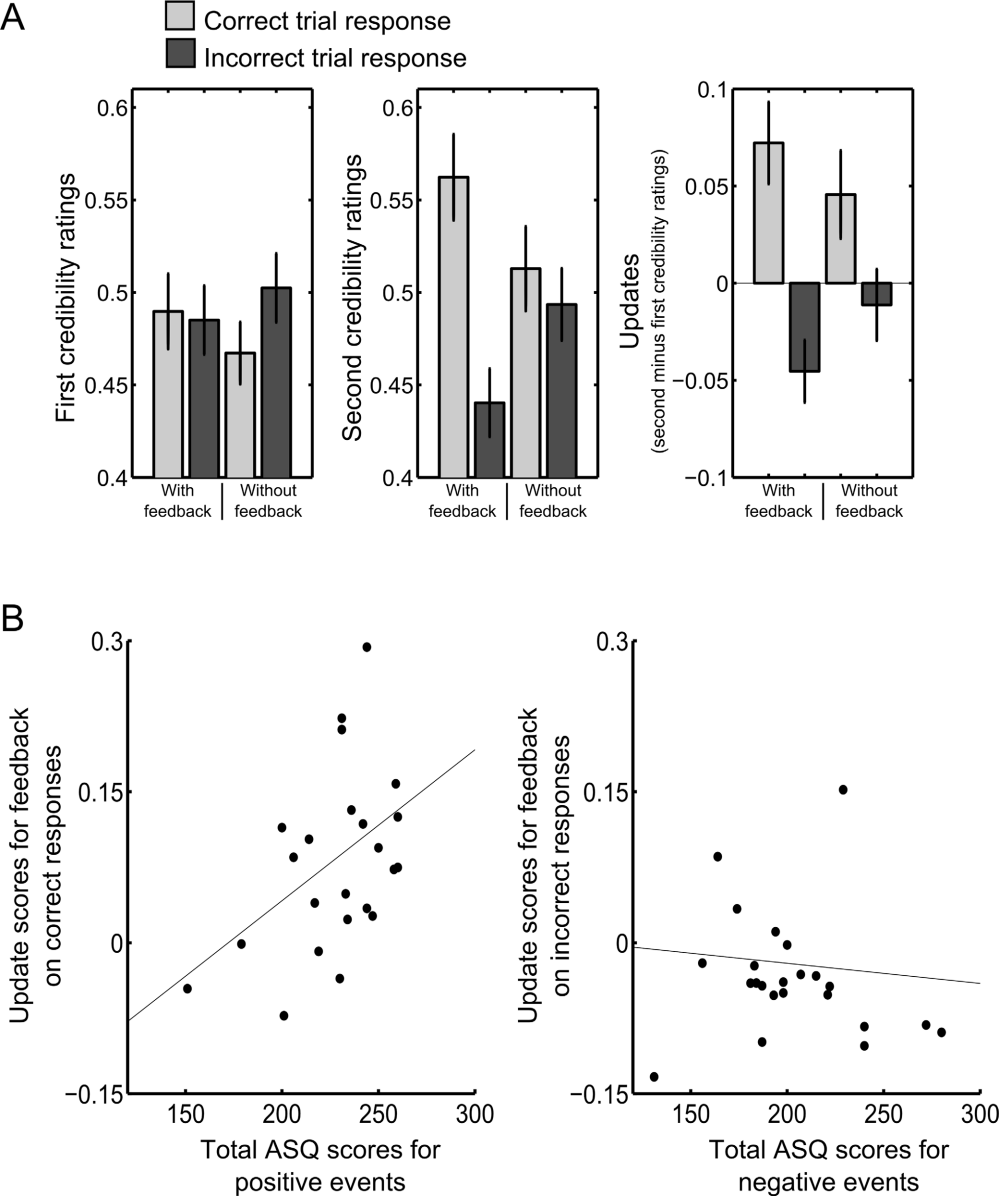
Ratings, updates, ASQ scores. (A) First credibility ratings (i.e., ratings before the emotion recognition task) lay around the midpoint of the scale. Second credibility ratings were higher after correct performance feedback than after incorrect performance feedback. A similar pattern emerged to a much lesser degree when no feedback was provided. For visualization, updates (i.e., the differences between second and first ratings) are plotted. (B) Relationship between total ASQ scores for positive events with updates after receiving feedback for correct performance (left) and no relationship between total ASQ scores for negative events with updates after receiving feedback for incorrect performance (right).

In line with our main hypothesis that participants should update toward the positive after feedback on correct task performance but toward the negative after feedback on incorrect task performance, we found a significant interaction on participants’ update scores in a Condition (feedback given / no feedback given) by Correctness (correct / incorrect task performance) ANOVA (both factors were within-subjects factors). For visualization see Fig 2A, for statistics see Table 1. Additionally, the main effect of Correctness but not the main effect of Condition reached significance. A follow-up *t*-test showed that when feedback was given updates following correct performance were higher than updates following incorrect performance (*t*(24) = -7.84; *p* < 10^−6^; all *p*-values Bonferroni corrected; in 24 out of 25 participants updates were higher after correct versus incorrect performance). The same pattern albeit much less strongly was observed for the condition in which no feedback was given (*t*(24) = -3.35; *p* < .05; in 19 out of 25 participants updates were higher after correct versus incorrect performance).

**Table 1:** Results from ANOVAs.

	Effect	*F*	*Df*	*P*	*η*_*p*_^*2*^
Updates (differences between first and second credibility ratings)	Condition	< 1.00	1, 24	n.s.	< .01
	Correctness	50.14	1, 24	< 10^−6^	.68
	Condition x correctness	8.56	1, 24	< .01	.26
First credibility ratings	Condition	< 1.00	1, 24	n.s.	< .01
	Correctness	2.44	1, 24	n.s.	.09
	Condition x correctness	5.58	1, 24	< .05	.19
Second credibility ratings	Condition	0.05	1, 24	n.s.	< .01
	Correctness	43.56	1, 24	< 10^−6^	.65
	Condition x correctness	33.21	1, 24	< 10^−5^	.58
Credibility ratings	Condition	0.00	1, 24	n.s.	.04
	Correctness	11.45	1, 24	< 0.005	.32
	Time	0.86	1, 24	n.s.	.04
	Condition x correctness	24.68	1, 24	< 10^−4^	.51
	Condition x time	0.19	1, 24	n.s.	< .01
	Correctness x time	48.78	1, 24	< 10^−6^	.67
	Condition x correctness x time	10.29	1, 24	< .005	.30
Number of trials	Condition	0.38	1, 24	n.s.	.02
	Correctness	0.05	1, 24	n.s.	< .01
	Condition x correctness	0.27	1, 24	n.s.	.01

Table listing the main and interaction effects of analyses of variance. All factors were within-subjects factors. Condition: feedback versus no feedback on performance (for each participant half of the trials were randomly assigned to the feedback and half to the no feedback condition). Correctness: correct versus incorrect performance (correctness depended on the individual participant’s performance in the given trial). Time: first versus second credibility ratings (participants rated the actors’ credibility before and after performing the emotion recognition task).

We also tested whether updates differed from zero when feedback was provided (i.e., whether there was a significant change from first to second credibility ratings). After correct performance feedback, updates were above zero indicating a change toward the positive (one-sample *t*-test against zero: *t*(24) = 3.39; *p* < .001). After incorrect performance feedback, they were below zero indicating a change toward the negative (*t*(24) = -2.82; *p* < .05). Importantly, when no feedback was given, updates did not differ from zero (all *p*’s > 0.2).

Taken together, our results indicate a positivity bias evidenced by changes in credibility ratings following performance feedback. Notably, these changes were significantly smaller in a control condition without feedback.

### Control analyses using linear mixed effects models confirmed the observed positivity bias

The above analyses on mean update scores per participant and condition treat participants as a random effect but video items as fixed effect and thus allow no generalization across video items. Therefore, we additionally used linear mixed effects models, which treated both participants and video items as random effects, to test whether the above described patterns for update scores held. This was indeed the case (see Table 2). Crucially, the Condition X Correctness interaction effect was significant for update scores as indicated by a *t*-value of 2.0 suggesting that participants updated toward the positive after feedback on correct task performance but toward the negative after feedback on incorrect task performance. When comparing the full model against the model without the fixed effect of the interaction the comparison favored the full model (χ^2^(1) = 16.30; *p* < 10^−4^; full model-BIC: 1034; reduced model-BIC: 1043; see Table 2 for linear mixed effects models on first and second ratings).

**Table 2:** Results from linear mixed effects models.

	Effect	*T*
Updates	Intercept	-0.77
	Condition	1.24
	Correctness	2.47
	Condition x correctness	-2.00
First ratings	Intercept	20.57
	Condition	0.57
	Correctness	-0.08
	Condition x correctness	-1.06
Second ratings	Intercept	11.04
	Condition	1.76
	Correctness	2.11
	Condition x correctness	-3.26

Table listing the main and interaction effects of linear mixed effects models. Condition: feedback versus no feedback on performance; Correctness: correct versus incorrect performance. See Table 1 for more details on the factors.

In sum, a linear mixed effects model including a random effects term for video items confirmed the interaction effect shown by the ANOVA on mean update scores per participant and condition.

### Control analyses showed that the non-random assignment of correct and incorrect trials did not drive the positivity bias

By design, the assignment of correct and incorrect performance feedback was not random across trials. That is, it depended on participants’ responses in the emotion recognition task. However, this did not drive our results. Firstly, there was no significant difference in the number of correctly solved items (*t*(24) = -0.130, *p* > .8) between conditions and thus participants’ performance in the recognition task resulted in a design that was balanced across participants. Secondly, participants’ first credibility ratings and subsequent correct responses were independent of each other (Fisher’s z transformed mean correlation overall participants: *r* = -0.035). Thirdly, across participants the mean number of correctly solved items did not correlate with the interaction effect or any of the differential mean update scores in the Condition (feedback given / no feedback given) by Correctness (correct / incorrect task performance) design (all *p*’s > .2, even without Bonferroni correction). Thus, more emotionally perceptive subjects were not more or less likely to denigrate actor credibility.

### Control analyses showed that positivity bias evident in the updates scores resulted from differences in second credibility ratings

Did update scores differ because of baseline differences that were already present before performance feedback (i.e., in the first credibility ratings)? To exclude this possibility, we analyzed first and second ratings separately. Ideally, ratings of the actors’ depictions should not differ between the conditions before the emotion recognition task. Instead, all relevant differences should emerge after the emotion recognition task. In our study, this was the case as detailed below.

In a Condition X Correctness ANOVA on first credibility ratings, the two main effects were not significant (all p’s > .1; see **Table 1**). We found a small but significant interaction effect at the .05-level (*F*(1,24) = 5.58; *p* = .027; see Fig 2A and Table 1). However, this interaction cannot account for the pattern observed in the updating scores—especially not for the cases in which participants received feedback. When performing individual *t*-tests between the four conditions no comparison survived Bonferroni correction. Only a trend emerged for those trials for which participants subsequently received no feedback: Participants rated stimuli which they subsequently answered incorrectly slightly higher than those which they subsequently answered correctly (*t*(24) = 2.64; *p* = .07). Additionally, mean first ratings did not differ from the midpoint of the rating scale, which was at 0.5 (in one-sample *t*-tests against 0.5 all *p*’s were > 0.2).

Importantly, the pattern described above for updates emerged in the Condition X Correctness ANOVA on second ratings: The interaction and the main effect of Correctness were significant while the main effect of Condition was not (see Table 1). Additionally, mean second ratings differed from the midpoint of the rating scale for items for which participants had received feedback (correct performance: *t*(24) = 2.70; *p* < .05; incorrect performance: *t*(24) = -3.23; *p* < .05) but not for items for which they had not received feedback (all *p*’s > 0.5).

The above control analyses show that particular effects related to their first credibility ratings cannot account for participants’ updating pattern. Notably, regression to the mean cannot explain updating since the first ratings did not differ across conditions and from the midpoint of the rating scale.

### Supplementary analyses show that reactions in the performance task followed the expected pattern

As common in many performance tasks, participants were faster for correctly versus incorrectly answered items in the emotion recognition task (RT correct items: mean = 3431 ms; *SD* = 617 ms; RT incorrect items: mean = 3833 ms, *SD* = 698 ms; *t*(24) = 7.87; *p* < 10^−7^). For two separate reasons, we did not analyze RTs for the credibility ratings. First, credibility ratings were given using a slider on a visual analogue scale and thus RTs depend on the distance between the initial and the final position of the slider. Second, credibility ratings constituted our main dependent variable of interest and we had no specific hypothesis for the RTs associated with credibility ratings.

### Attributional style questionnaire (ASQ)

To compare our assessment of the positivity bias in the current task with a previous measure of the positivity bias in attribution, participants completed the ASQ, which is the most widely used measure [7]. In line with previous reports the total score was higher for positive than for negative events (see **Table 3**). To explore the relationship between updating behavior in our task and attributional style as measured by the ASQ, we correlated the total ASQ score for positive events with updates after receiving feedback for correct performance and the ASQ score for negative events with updates after receiving feedback for incorrect performance, respectively. The former (Pearson’s *r* = .45; *p* = .06) but not the latter (*p* > .5) showed a trend for a correlation after Bonferroni correction and the strength of the two correlations differed significantly as determined by Steiger’s *z* (*z* = 2.05; *p* < .05; see Fig 2B).

**Table 3:** Attributional style questionnaire (ASQ).

	Positive events		Negative events		*t*	*df*	*p*	Cohen’s *d*
	Mean	SD	Mean	SD				
Internality	72.9	9.3	70.0	13.9	< 1.00	22	n.s.	.18
Stability	74.1	9.5	63.1	13.8	3.97	22	< .001	.83
Globality	81.5	15.8	70.0	16.7	3.03	22	< .01	.63
Total	228.1	27.2	202.4	34.8	2.86	22	< .01	.60

*T*-tests compared the scores for positive and negative events.

A positive correlation between updates after positive feedback in our task and the total ASQ scores for positive items suggests that participants with a greater tendency to rate the credibility of the actors higher after receiving positive feedback on the task were more likely to attribute positive events to internal, global and stable causes.

## Discussion

The aim of this study was to show a positivity bias in attributions using a decision-making task. In this task, participants rated the credibility of the depiction of different facial emotions by actors before and after receiving performance feedback on an emotion recognition task with the same items. This setup overcomes important drawbacks inherent to classic descriptions of the positivity bias in attributions: Our task assess attribution implicitly following real (and not imaginary) performance feedback and we obtain baseline ratings of the stimuli. Crucially, we show that attribution processes are relevant for decision-making. In accordance to our hypotheses, participants changed their beliefs of the task-relevant stimuli depending on the performance feedback they received. That is, participants evaluated the stimuli more positively after having received feedback on correct task performance and more negatively after having received feedback on incorrect task performance. This constitutes evidence for a self-serving bias because feedback on incorrect performance—which according to attribution theory is attributed to external, unstable, and to specific causes—leads to a downgrading of the stimuli (i.e., the actors’ credibility). In contrast, feedback on correct performance leads to a change towards the positive. A similar pattern emerged to a lesser degree when no feedback was given, which may indicate that participants can to some degree infer whether their performance was correct or incorrect even without receiving explicit performance feedback. Updates after receiving positive feedback were related at trend level to the scores for positive items of the ASQ, which constitutes the most commonly used explicit measure of attributional style. We did not find a significant relationship between updates after receiving negative feedback and ASQ scores for negative items.

### Merits of attribution theory

In our view, merits of attribution theory rest on two of its facets. First, it points out that different types of causes can determine outcomes such as task performance. In contrast, models in the decision neuro(science) literature often abstract away from the potential multi-causality of real life events [30,31]. Second, the empirical evidence that meta-cognitive assessments of causality tend to be self-serving nicely link attribution theory to a wider literature on self-related positivity biases [52–54].

### Overcoming the drawbacks of previous investigations of the positivity bias in attribution

Our procedure overcomes three important drawbacks of previous demonstrations of the self-serving bias in attribution: First, it does not include explicit meta-cognitive assessments of causality, which are difficult to generalize to more implicit real life settings. Rather it is an indirect measure of attribution processes, in that it measures feedback related changes in the evaluation of the actors’ credibility. Here, participants are neither explicitly asked to infer the causes for their correct or incorrect performance after the emotion recognition task nor are they prompted to take the feedback on the independent emotion recognition task into account when judging the actors’ performance a second time. This difference with respect to traditional meta-cognitive assessments of attribution styles ensures that participants *implicitly* shift their ratings towards the positive after receiving feedback on correct performance and towards the negative after receiving feedback on incorrect performance.

Second, performance feedback was given on a narrowly framed emotion recognition task, in which participants received both correct and incorrect feedback. Previous studies have often used loosely defined complex events or even imagined events (as for example in the ASQ). Our study design rules out the possibility that participants just imagine hypothetical events in a biased way, which would constitute a bias in imagination but not a bias in causality attributions.

Third, we elicited participants’ beliefs before and after performance feedback. Thereby, we exclude the possibility that self-serving attributions appear because of initial properties of the stimuli, which cannot be controlled for with a single assessment.

### Relation to classical assessments of attribution

Similar to previous studies [16], we enhanced the self-relevance of the task by telling participants that they would receive a scoring of their performance with respect to others at the end of the study. To further increase the study’s ecological validity and saliency to participants, the task comprised dynamic facial emotional expressions of a large number of male and female actors of varying ages. We ensured optimal task difficulty (i.e., avoiding ceiling and floor effects) by including a large number of emotional expressions. We acknowledge, however, that in contrast to traditional, direct assessments of causal attribution, our task design cannot distinguish between different types of self-attributions. Nevertheless, the correlation between updates after positive feedback and the total ASQ scores for positive items suggests that the attribution processes in our task and in the most commonly used traditional measure are related, at least to some degree.

### Relation to other positivity biases in feedback processing

Conceptually related positivity biases have been described when participants receive self-relevant feedback on their future or on their character traits [33,35]. Desirable feedback leads to a greater belief update than undesirable feedback. Here, we provide evidence that the processing of performance feedback can result in a similarly biased updating pattern as described by the self-serving attribution bias: The evaluated credibility of actors depicting facial emotions changes toward the positive after desirable performance feedback (i.e., feedback indicating that the depicted emotions were correctly recognized). The inverse was true after undesirable feedback (i.e., feedback on incorrect emotion recognition).

In the current task, participants could not choose whether to receive feedback or not. Previous research indicates that feedback seeking can in itself be positively biased [55]. After failure but not after success feedback for an unrelated anagram task, humans seek more positive than feedback negative about their personal abilities. Thereby, they may avoid having to make attributions for negative feedback. Such a pattern could be easily tested in version of the current task, in which participants can decide after each emotion recognition question whether they get feedback or not. Taken together, these findings highlight the question if all of these biases can be integrated into a common account.

### Motivational accounts and their relation to psychiatric disorders

As indicated by the label “self-serving bias,” usually motivational accounts are brought forth to explain the empirically observed pattern that healthy individuals attribute positive outcomes (including correct performance) internally but negative outcomes (including incorrect performance) externally [1,2,8,56]. Motivational accounts are often highlighted because of their relevance for understanding maladaptive behaviors and beliefs in psychiatric populations. In line with this notion, psychiatric populations, especially depressed patients, typically show a less biased attribution pattern [7,10]. Depressive patients also show a reduction in positively biased updating for future life events [34,57]. Future studies would ideally use a similar design as we did here to establish the relevance of the implicit self-serving bias for psychiatry.

### Adaptiveness of positively biased attribution

So far, the questions whether and why biases such as a positively biased attribution pattern can be adaptive have been notoriously difficult to address ([58], for related discussions see [59–61]). One possibility is that a positively biased assessment of causality leads individuals to spend their scarce resources on solvable tasks for which they can easily improve their abilities. In this sense, the adaptive nature of the bias strongly depends on individuals’ current environment. Extensions of our task design offer the possibility to manipulate how much participants benefit from positively biased performance feedback processing. We predict that in such cases the magnitude of the bias would depend on its adaptive nature.

Interestingly, previous research has shown that in some cases stigmatized groups tend to attribute negative feedback in an adaptive way; that is they think that others rated them negatively because of stigma-related stereotypes (see [62] for an example where African Americans receive feedback on an essay from a Caucasian evaluator and attribute negative feedback to stereotypes against African Americans). Based on these findings, we predict that groups with a stereotype of poor emotion recognition skills may exhibit an altered attribution pattern in our task. For example, they may attribute negative performance feedback to the stereotype and may therefore not update their credibility ratings following negative feedback.

### Challenges to the motivational account

Despite the widespread motivational interpretation of internal attributions of correct performance versus external attributions of incorrect performance, the “self-serving” nature of this pattern has been challenged since its early demonstrations [58]. Contestants have argued that the cognitive explanations are better suited to account for the empirically observed causality attributions [56,58]. Cognitive explanations include assertions that positive outcomes are attributed internally because they are more consistent with individuals’ expectations or their actions. Alternatively, individuals may have different standards of proof for correct and incorrect performance. Although a consensus in the literature seems to be that motivational and cognitive factors are often intertwined and are not mutually exclusive [8,56], we still argue that efforts should be made more precisely characterize their respective contributions. In this regard, our implicit assessment shows that a positivity bias emerges even in the absence of explicit assessments of causality. In our view, the fact that participants’ initial ratings of items were similar, independent of whether they subsequently received feedback on correct or incorrect performance, makes explanations relating to individuals’ expectations unlikely. That is, participants could not anticipate a certain performance feedback during their first assessment. Additionally, we hold it unlikely that participants explicitly considered different standards of proof for positive and negative performance since they were not prompted to provide explicit causality statements. Furthermore, future studies could extend our task design by varying the reliability of the information provided or by assessing participants’ confidence. This may help to dissociate effects related to motivational influences from effects related to information processing.

### Limitation

We used a visual analogue scale with a slider that started in the midpoint of the scale to elicit participants’ credibility ratings. This may have anchored participants’ responses to the midpoint of the scale. In addition, the use of a slider makes reaction times dependent on the difference between starting and ending positions and we therefore do not report exploratory analyses of reaction times of participants’ ratings. We preferred a constant anchoring to the midpoint for all trials to a varying anchoring to a random point on the scale. The latter would have necessitated an elaborate dissection of the trial-by-trial differences in anchoring in order to obtain corrected estimates of participants’ credibility ratings. Ideally, our results should be replicated in a free response format.

## Conclusions

To summarize, our study shows that the self-serving attribution bias emerges in an task akin to those used in the decision-making literature. We showed that positive and negative performance feedback on an emotion recognition task changes the individual’s initial credibility rating for a displayed emotion in a positive or negative way, respectively. We found evidence for a relation between our implicit measure and one of the most commonly used explicit measures for positive events. We strongly expect the observed pattern to hold over a variety of tasks and stimulus classes as long as task and stimuli are perceived as self-relevant and reasonably easy, thereby avoiding that participants merely attribute performance to task difficulty. The wider implication of our study is that it links attribution theory to theories of feedback processing in decision-making. As predicted by attribution theory, whether individuals incorporate past performance feedback into their future decisions, depends on the extent to which they hold themselves responsible for the feedback they received.

## Supporting Information

S1 Table Stimuli details. Description of the video items used in the task.
(DOCX)
