# Supplementary material for: Performance Feedback Processing Is Positively Biased As Predicted by Attribution Theory
Source: PLoS One. 2016 Feb 5;11(2):e0148581. doi: 10.1371/journal.pone.0148581 (PMC4743912; doi:10.1371/journal.pone.0148581)
Supplement: S1 Table — Description of the video items used in the task. (DOCX) [file pone.0148581.s001.docx]

**S1 Table. Stimuli details.**

|  | List 1 emotions (n=48) | | | List 2 emotions (n=48) | | |
| --- | --- | --- | --- | --- | --- | --- |
| n | Target emotion | *actor number* | *actor gender* | Target emotion | *actor number* | *actor gender* |
| 1 | angry | 1 | male | angry | 19 | male |
| 2 | angry | 8 | male | apologetic | 21 | female |
| 3 | apologetic | 5 | female | apologetic | 24 | female |
| 4 | apologetic | 10 | female | bored | 3 | female |
| 5 | apologetic | 13 | male | bored | 7 | female |
| 6 | apologetic | 15 | male | bored | 22 | female |
| 7 | bored | 12 | female | compassionate | 4 | male |
| 8 | bored | 23 | male | compassionate | 7 | female |
| 9 | compassionate | 3 | female | compassionate | 12 | female |
| 10 | compassionate | 6 | female | compassionate | 15 | male |
| 11 | confused | 5 | female | compassionate | 22 | female |
| 12 | confused | 10 | female | confused | 6 | female |
| 13 | contemptuous | 13 | male | confused | 11 | female |
| 14 | contemptuous | 17 | male | confused | 19 | male |
| 15 | desperate | 6 | female | confused | 24 | female |
| 16 | disappointed | 10 | female | contemptuous | 1 | male |
| 17 | disappointed | 18 | male | contemptuous | 4 | male |
| 18 | disappointed | 21 | female | desperate | 26 | female |
| 19 | doubtful | 3 | female | disappointed | 13 | male |
| 20 | doubtful | 24 | female | disappointed | 20 | male |
| 21 | embarrassed | 8 | male | disappointed | 26 | female |
| 22 | embarrassed | 11 | female | disappointed | 27 | female |
| 23 | envious | 3 | female | doubtful | 8 | male |
| 24 | envious | 14 | female | embarrassed | 9 | male |
| 25 | envious | 17 | male | embarrassed | 10 | female |
| 26 | fearful | 5 | female | forgiving | 8 | male |
| 27 | fearful | 18 | male | forgiving | 25 | female |
| 28 | fearful | 20 | male | frustrated | 8 | male |
| 29 | forgiving | 22 | female | frustrated | 21 | female |
| 30 | forgiving | 16 | male | frustrated | 23 | male |
| 31 | frustrated | 4 | male | guilty | 2 | female |
| 32 | frustrated | 25 | female | guilty | 18 | male |
| 33 | frustrated | 17 | Male | guilty | 26 | female |
| 34 | guilty | 16 | male | horrified | 7 | female |
| 35 | guilty | 9 | male | horrified | 9 | male |
| 36 | horrified | 12 | female | hurt | 1 | male |
| 37 | hurt | 2 | female | hurt | 15 | male |
| 38 | hurt | 22 | female | jealous | 7 | female |
| 39 | jealous | 19 | male | jealous | 17 | male |
| 40 | melancholic | 9 | male | offended | 18 | male |
| 41 | melancholic | 14 | female | offended | 22 | female |
| 42 | offended | 1 | male | sad | 5 | female |
| 43 | offended | 2 | female | sad | 27 | female |
| 44 | offended | 17 | Male | shocked | 2 | female |
| 45 | sad | 24 | female | worried | 1 | male |
| 46 | shocked | 7 | female | worried | 15 | male |
| 47 | worried | 8 | male | worried | 17 | male |
| 48 | worried | 20 | male | worried | 24 | female |
